# Noncoding RNAs involved in the STAT3 pathway in glioma

**DOI:** 10.1186/s12935-021-02144-y

**Published:** 2021-08-23

**Authors:** Zheng Bian, Wei Ji, Bin Xu, Zhengyuan Huo, Hui Huang, Jin Huang, Jiantong Jiao, Junfei Shao, Xiaolu Zhang

**Affiliations:** Department of Neurosurgery, Wuxi People’s Hospital of Nanjing Medical University, Wuxi, People’s Republic of China

**Keywords:** glioma, miRNA, lncRNAs, circRNAs, STAT3

## Abstract

Glioma is the most common malignant primary brain tumour in adults. Despite improvements in neurosurgery and radiotherapy, the prognosis of glioma patients remains poor. One of the main limitations is that there are no proper clinical therapeutic targets for glioma. Therefore, it is crucial to find one or more effective targets. Signal transducer and activator of transcription 3 (STAT3) is a member of the STAT family of genes. Abnormal expression of STAT3 is involved in the process of cell proliferation, migration, invasion, immunosuppression, angiogenesis, dryness maintenance, and resistance to radiotherapy and chemotherapy in glioma. Therefore, STAT3 has been considered an ideal therapeutic target in glioma. Noncoding RNAs (ncRNAs) are a group of genes with limited or no protein-coding capacity that can regulate gene expression at the epigenetic, transcriptional and posttranscriptional level. In this review, we summarized the ncRNAs that are correlated with the ectopic expression of STAT3 in glioma.

## Introduction

Glioma is the most common primary intracranial malignant tumour. Despite the great progress that has been made in neurosurgical technology, radiotherapy and chemotherapy, the prognosis of glioma patients is still poor [1]. Particularly in glioblastoma (GBM), the 5-year survival rate of patients is still less than 5%, and the median survival time is less than 1 year [2]. One of the main reasons for the poor prognosis of glioma is that there are no specific and effective therapeutic targets in clinical practice. Therefore, elucidating the molecular mechanism underlying tumorigenesis and identifying novel therapeutic targets are essential for the treatment of glioma.

Noncoding RNAs (ncRNAs) are a group of genes with limited or no protein-coding capacity [3]. Based on their length, ncRNAs are divided into long noncoding RNAs (> 200 nt) and small noncoding RNAs. Small noncoding RNAs can be divided into small nuclear RNAs (snRNAs), small nucleolar RNAs (snoRNAs), microRNAs (miRNAs), and piRNAs. Noncoding RNAs account for 60% of human transcripts, and they were previously thought to be genomic “dark matter” [4]. However, an increasing number of studies have found that noncoding RNAs play important roles in the physiological and pathological processes of various human diseases, including tumours [5, 6]. The role of noncoding RNAs in glioma has also been widely investigated. Studies have found that noncoding RNAs are involved in a series of processes, such as the occurrence, development, and treatment resistance of gliomas, and have become a potential prognostic, diagnostic indicator, and treatment target [7, 8].

Signal transducer and activator of transcription 3 (STAT3) is a member of the signal transducer and activator of transcription (STAT) protein family, which can be activated by a variety of cytokines or growth factors [9]. STAT protein family members contain an amino end domain, coiled-coil domain, DNA-binding domains (DBD), Src homology 2 domain (SH2) domain, tyrosine activation domain and transcription activation domain (TAD). The activation of STAT3 can be induced by epidermal growth factor receptor (EGFR), JAK2, and other tyrosine kinases activated by epidermal growth factor (EGF), leukaemia inhibitory factor (LIF), and other cytokines [10]. Upon activation, STAT3 can form homodimers or heterodimers (STAT1/3) and enter the nucleus. Then, homodimers or heterodimers interact with the corresponding target gene promoter to activate gene transcription and translation. Janus kinase (JAK), acting as a transcription factor, plays an important role in the activation of STAT3. The classic signalling cascade of the JAK/STAT pathway involves the dimerization of receptor molecules caused by the cytokine binding to its receptor leading to the coupling of JAK receptors, which are brought into close proximity and then activated by interactive tyrosine phosphorylation. Following JAK activation, JAKs catalyse the tyrosine phosphorylation of the receptor itself and form corresponding STAT docking sites. Upon the activation of JAKs, STAT3 binds to receptors through the SH2 domain and is phosphorylated [11].

The expression and activation of STAT3 are precisely regulated, including its proteasomal degradation and the activation-inactivation mechanism that regulates the transient activation of STAT3 [12]. It is clear that the suppressor of cytokine signalling (SOCS) protein family and the protein inhibitor of activated STAT (PIAS) protein family are important negative regulators of the STAT signalling pathway. Among them, SOCS can inhibit STAT signal transduction by binding and inhibiting JAKs [13]. PIAS only binds to activated STAT dimers. By recruiting acetylase, it promotes the SUMO (small ubiquitin-like modifier) modification of STAT and induces dimerization and dissociation to inhibit the DNA binding and transactivation ability of STAT3 [14]. In normal cells, the activation of STAT3 is transient, but in gliomas, due to the lack of upstream regulatory signals and negative regulatory mechanisms, STAT3 is constitutively highly expressed and activated. The high expression and activation of STAT3 play an important role in gliomas. For example, in vivo and in vitro experiments have shown that c-myc, CCND1, Bcl-2, BCL-XL, Survivin, etc., are the direct target genes of STAT3 in the regulation of glioma cell proliferation [15, 16]. STAT3 regulates the migration and invasion of glioma cells by targeting MMP2, MMP9, SNAIL, etc. [17]. STAT3 regulates angiogenesis by targeting VEGF [18]. In addition, STAT3 regulates the activity and proliferation of T cells and the function of dendritic cells to participate in tumour immunity [19, 20]. STAT3 also plays a significant role in the resistance to radiotherapy and chemotherapy of glioma [21, 22].

In glioma, ncRNAs can play an important role in upstream signals and mechanisms to regulate the expression and activation of STAT3. In addition, STAT3, acting as a transcription factor, can directly target and then regulate the expression of ncRNAs. To further understand the occurrence of glioma and provide new ideas for the development of STAT3-targeted tumour therapy, this article mainly summarizes the ncRNAs involved in the STAT3-mediated signalling pathway in glioma.

## MiRNAs involved in the STAT3 signalling pathway

MiRNAs are endogenous, noncoding small RNAs with an approximate length of 20–22 nucleotides that mainly participate in regulating gene transcription, translation, or epigenetic processes. They are widely found in eukaryotes, with a high degree of conservation, cell and tissue specificity and timing [23]. The production of miRNAs is a multistep process and involves the canonical biogenesis pathway. In the nucleus, the miRNA gene is transcribed into a primary miRNA transcript (pri-miRNA) that is 300–1000 base pairs (bp) in length by RNA polymerase II [24]. Mediated by the RNase III Drosha, pri-miRNAs are cleaved to pre-miRNAs to a length of approximately 70 bp and contain a stem-loop structure [25]. Then, pre-miRNAs are transported from the nucleus to the cytoplasm by the Ran-GTP-dependent transporter exportin 5 (XPO5) [26]. In the cytoplasm, Dicer, a double-stranded RNA-specific endonuclease, and TAR RNA-binding protein (TRBP) recognize pre-miRNAs. Pre-miRNAs are further processed by Dicer to generate mature double-stranded miRNAs with a length of 20–22 nucleotides. To regulate gene expression, mature double-stranded miRNAs unwind; then, one of the strands is degraded, and the other is integrated into the RNA-induced silencing complex (RISC) and guides it to the target mRNA. Finally, the complex binds to its target mRNA, inhibiting gene expression [27, 28].

**Fig. 1:**
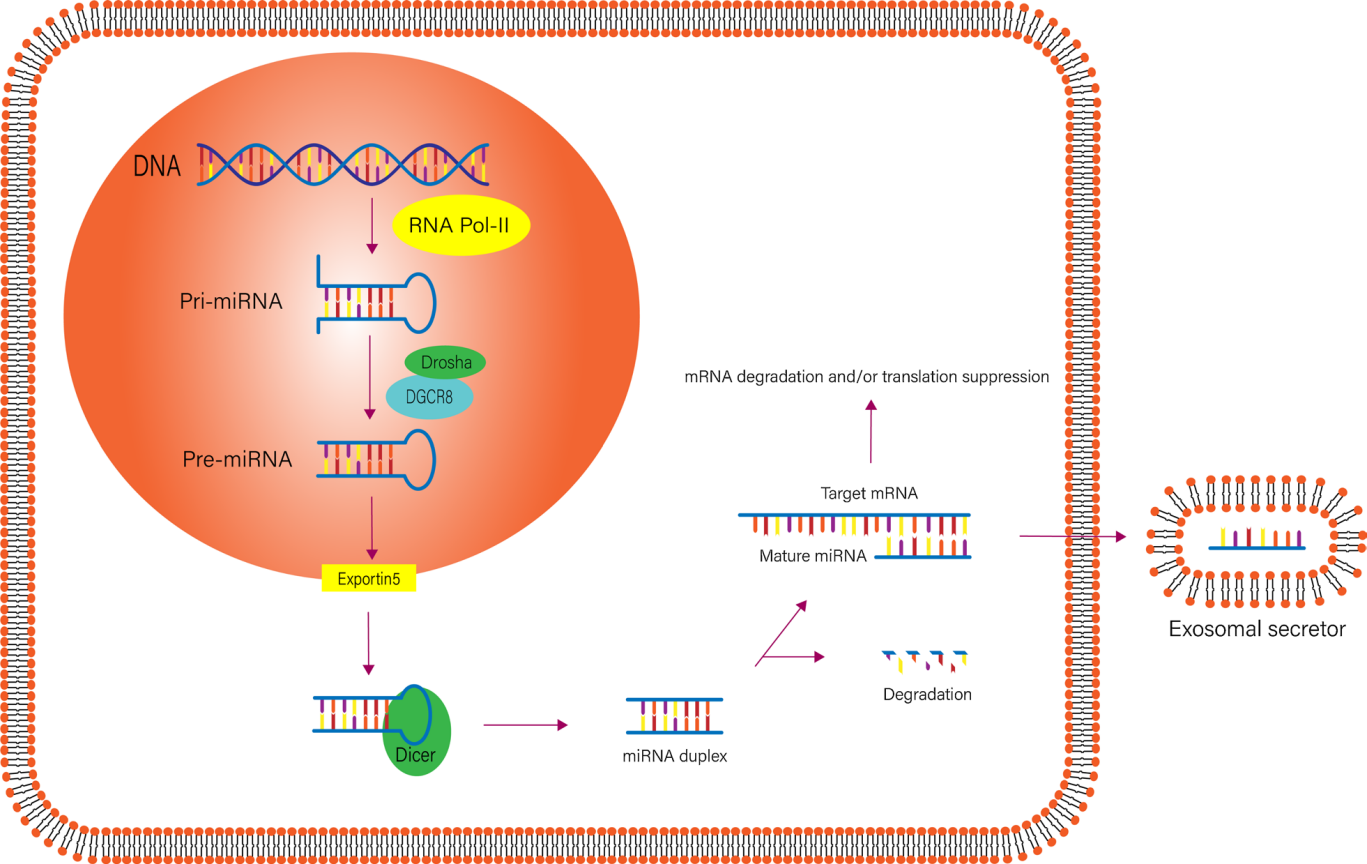
Biogenesis of miRNAs. In the nucleus, the miRNA gene is transcribed into a pri-miRNA by RNA polymerase II, and then pri-miRNAs are cleaved to pre-miRNAs by RNase III Drosha. Pre-miRNAs are transferred from the nucleus to the cytoplasm by XPO5. In the cytoplasm, pre-miRNAs are further processed to mature double-stranded miRNAs by Dicer. The mature double-stranded miRNAs unwind, and then one of the strands is degraded and the other is integrated into the RISC, which then guides it to the target mRNA. In addition, miRNAs can be secreted and regulate target gene expression via the exosomal pathway

MiRNAs play important regulatory roles in eukaryotes by binding to corresponding mRNA transcripts and then leading to their degradation and/or translation inhibition at the mRNA level [29]. Studies have shown that miRNAs can function not only as a single entity but also in the form of gene clusters. A miRNA gene cluster refers to a gene group composed of two or more miRNAs that are closely adjacent to each other on a chromosome. The miRNAs arranged in clusters may have a homologous relationship, or they may not show any homology. Meanwhile, the expression patterns of miRNAs arranged in clusters are highly consistent and have coordinated regulatory effects [30]. The most common mechanism underlying gene expression regulation by a mature miRNA is through its interaction with the 3′-untranslated region (UTR) of the target mRNA or the complementary sequence of the coding region leading to mRNA degradation or translation inhibition [31, 32]. In addition, studies have shown that miRNAs could also interact with the 5′-UTR, coding sequences and the promoter regions of their target genes. MiRNA binding to the 5'-UTR of the target gene will promote its transcription, which is the opposite of what occurs when a miRNA binds to the 3'-UTR [33]. In addition, in recipient cells, miRNAs can be secreted and regulate target gene expression via the exosomal pathway [34] (Fig. 1).

An increasing number of studies have shown that miRNAs are involved in the occurrence and development of gliomas by acting alone or in the form of gene clusters. These miRNAs may regulate the expression of nearly one-third of the genes in the genome. Different miRNAs are involved in distinct pathophysiological processes, including the proliferation, migration, invasion, apoptosis, radiotherapy, and chemotherapy resistance of glioma cells. The occurrence of glioma is closely related to the increase in the expression of tumour-promoting miRNAs and the decrease in the expression of tumour-suppressing miRNAs [35]. In addition, due to the stability of miRNA structure, its role as a diagnostic and prognostic indicator of glioma is receiving increasing attention [36]. The use of miRNA mimics and anti-miRNA oligonucleotides to treat tumours in animals has achieved clear results. Therefore, miRNAs are also considered potential targets for tumour therapy. The miRNAs related to the STAT3 signalling pathway in gliomas can be divided into the following types as shown in Table 1.

**Table 1.** MiRNAs involved in STAT3 signaling pathway

MicroRNA	Up-/down-regulation	Role in glioma	Biological functions	Target gene	Refs.
miR cluster MC‐let‐7a‐1 ~ let‐7d	Down	Tumor suppressor	Inhibit proliferation, promote apoptosis, and cell autophagy	STAT3	[40]
miR-506	Down	Tumor suppressor	Inhibit proliferation, migration, and invasion	STAT3	[42]
miR-181d	Down	Tumor suppressor	Inhibit migration and invasion	STAT3/STAT5A	[43]
miR-29b	Down	Tumor suppressor	Inhibit chemoresistance	STAT3	[45]
miR-519a	Down	Tumor suppressor	Inhibit proliferation, migration, invasion, and chemoresistance	STAT3	[46, 47]
miR-124	Down	Tumor suppressor	Inhibit proliferation, promote apoptosis, and immune therapeutics	STAT3/SAMD4	[48, 56]
miR‐221/222 cluster	Up	Oncogene	Promote migration, invasion, proliferation, and angiogenesis	SOCS3	[50]
miR-30	Up	Oncogene	Promote tumorigenesis	SOCS3	[51]
miR-125b	Up	Oncogene	Promote chemoresistance	PIAS3	[53]
miR-133a	Down	Tumor suppressor	Inhibit proliferation, migration, and invasion, promote apoptosis	CTGF	[55]
miR-148a	Up	Oncogene	Promote migration, invasion	CADM1	[58]
miR-876-3p	Down	Tumor suppressor	Inhibit proliferation, migration, and invasion	KIF20A	[60]
miR-26a	Up	Oncogene	Promote chemoresistance, migration, invasion, proliferation	AP-2α	[62]
miR-6743-5p	Up	Oncogene	Promote proliferation, inhibit apoptosis	GRIM-19	[63]
miR-1246	Up	Oncogene	Promote proliferation, migration, and invasion	TERF2IP	[65]
miR-125b/miR-20b	Up	Oncogene	Promote proliferation	FZD6/ALDH1A3	[67]
miR-21	Up	Oncogene	Promote proliferation and invasion	RECK	[68, 69, 70, 71]
miR-182-5p	Up	Oncogene	Promote proliferation, migration, and invasion	PCDH8	[72]
miR-30b-3p	Up	Oncogene	Promote proliferation, chemoresistance, and inhibit apoptosis	RHOB	[73]
miR-218	Down	Tumor suppressor	Inhibits proliferation	PDGFRα	[74]
miR-34a	Down	Tumor suppressor	Inhibit migration and invasion	–	[75]
miR-184	Up	Oncogene	Promote proliferation and invasion	–	[76]
miR-203	Down	Tumor suppressor	Radiosensitivity	–	[77]
miR-31	Down	Tumor suppressor	Promote apoptosis	–	[78]
miR-27b	Up	Oncogene	Promote invasion, proliferation, inhibit apoptosis	–	[79]
miR-302/367	Down	Tumor suppressor	Inhibit proliferation, migration	–	[80]

### MiRNAs directly targeting STAT3

In GBM, on the one hand, miRNAs can directly target STAT3 to regulate the expression of related proteins in the STAT3 pathway, thereby affecting tumour cell function [37]. In terms of glioma cell proliferation, the B-cell lymphoma/leukaemia-2 gene (bcl-2) plays an important role in tumour cell anti-apoptosis, promotes tumour cell proliferation, and inhibits tumour cell apoptosis [38]. LC3 is a significant marker of autophagy, while P62 acts as a substrate of ubiquitination [39]. MiRNA clusters let-7a-1 ~ let-7d (including let-7d, let-7f-1 and let-7a-1) can directly target and inhibit STAT3 to promote apoptosis and autophagy of glioma cells. A study confirmed that STAT3 was the direct target gene of these three let-7 miRNA dual luciferase reporters. Then, cell fluorescence staining and experiments in mouse models confirmed that miRNA clusters of let-7a-1 ~ let-7d acted as tumour suppressors in glioma [40]. In terms of migration and invasion, the growth and metastasis of GBM depend on the expression of matrix metalloproteinase 2 (MMP2). Overexpression of MMP2 promotes the metastasis and spread of various tumours and is the most important molecule that directly promotes tumour metastasis [41]. MiR-506 directly targets STAT3 to exert a tumour suppressor effect. Functional experiments have shown that overexpression of miR-506 could inhibit the migration and invasion of glioma cells and reduce the protein expression levels of MMP2, cyclin D1 and Bcl-2 [42]. Interestingly, by using Schrodinger PyMOL 2.3 molecular docking and visualization software, it has been demonstrated that miR-181d interacts with and binds directly to STAT3 or STAT5A. Upregulating miR-181d inhibits GBM cell migration, invasion, and tumour growth in GBM mouse models by suppressing STAT3 or STAT5A expression [43].

On the other hand, miRNAs can directly target STAT3 and function as therapeutic tools for GBM treatment. In terms of chemotherapy resistance, temozolomide (TMZ) is a standard chemotherapy drug for GBM multiforme. However, the occurrence of TMZ resistance is increasing rapidly in clinical treatment. In recent years, miRNAs have been confirmed to act as important regulators in the process of drug resistance [44]. Overexpression of miR-29b can enhance the sensitivity of TMZ-resistant glioma cells to TMZ by inhibiting the expression of STAT3 [45]. MiR-519a functions as a tumour suppressor by directly targeting STAT3 in GBM. In vitro studies have shown that miR-519a can increase the sensitivity of glioma cells to TMZ by promoting autophagy, while in vivo experiments in nude mice have also proven that miR-519a enhances the therapeutic effect of TMZ on glioma cells. In addition, the decreased expression of miR-519a is related to shorter tumour-free survival and overall survival rates. In terms of immune therapeutics, STAT3 signalling has been shown to be a key regulator of the microglia/macrophage-mediated immune response [46, 47]. MiR-124 can directly target STAT3 in glioma cells to inhibit their proliferation and promote cell apoptosis. Studies have found that miR-124 plays a therapeutic role in genetically engineered mouse models expressing STAT3 and strengthens the immune response mediated by T cells [48].

### MiRNAs regulate the expression of genes upstream of STAT3

It has been reported that p-STAT3 is key for the function of STAT3. P-STAT3 forms homodimers or heterodimers (STAT1/STAT3) through mutual phosphotyrosine-SH2 domain interactions, enters the nucleus, and regulates the transcription of target genes. Regarding the negative regulation of STAT3 activation, the SOCS and PIAS protein families play important roles. Suppressor of cytokine signalling 3 (SOCS3) belongs to the SOCS family and harbours an SH2 domain, which can bind to the active region of JAKs through the SH2 domain, preventing it from phosphorylating STAT3 [49]. RT-qPCR found that miR-221 and miR-222 are highly expressed in glioma cell lines. Both in vivo and in vitro experiments have shown that inhibition of the miR-221/222 cluster can inhibit cell migration, invasion, proliferation, and angiogenesis. Therefore, miR-221 and miR-222 are considered to act as oncogenes in gliomas. In terms of the mechanism, the miR-221/222 cluster directly targets SOCS3 and inhibits its expression, resulting in an increase in the protein level of p-STAT3 [50]. In addition, miR-30 was identified as a potential oncogene in GBM. MiR-30 can also increase the protein expression of p-STAT3 to promote tumorigenesis by targeting SOCS3 [51]. Protein inhibitor of activated STAT3 (PIAS3) binds to an activated homodimer or heterodimer (STAT1/STAT3) and then blocks STAT3 DNA-binding activity [52]. In glioblastoma stem cells (GSCs), the expression level of miR-125b is high. A miR-125b inhibitor enhanced the invasion-prevention activity of temozolomide in GSCs by targeting PIAS3, which then reduced STAT3 transcriptional activity and subsequently decreased the expression of matrix metalloproteinases MMP2 and MMP9 [53].

In addition to SOCS3 and PIAS3, miRNAs can regulate growth factor/cytokine receptors, which are common ways to activate STAT3. Connective tissue growth factor (CTGF) is related to the occurrence and development of tumours; participates in cell proliferation, development, adhesion, migration, and angiogenesis; and predicts prognosis [54]. MiRNA-133a, which is expressed at low levels in human glioma tissue and glioma cells, targets CTGF and then regulates the JAK/STAT signalling pathway by acting as a tumour suppressor gene [55]. Transforming growth factor-β (TGF-β), which functions as an oncogene in glioma, can activate the Jak/Stat3 pathway in a Smad-dependent manner. In addition to directly targeting STAT3, miR-124 regulates the protein levels of STAT3 and p‑STAT3 to inhibit cell proliferation by targeting Smad4 [56]. Additionally, it has been reported that cell adhesion molecule 1 (CADM1) can interact with HER2 and Itga6b4 to reduce STAT3 pathway activity [57]. MiR-148a targets CADM1 to regulate STAT3 pathway activity and promote cell metastasis [58].

MiRNAs also regulate other proteins upstream of STAT3 signalling pathways. Kinesin family member 20A (KIF20A), a member of kinesin superfamily 6, plays a significant role in the occurrence and development of tumours [59]. Functional experiments have shown that miR-876-3p inhibits cell proliferation, migration, and invasion. MiR-876-3p also acts as a tumour suppressor in a nude mouse model. Mechanistic experiments have proven that miR-876-3p inhibits the JAK2/STAT3 signalling pathway by targeting KIF20A [60]. AP-2α, a central member of the AP-2 family, can function as a tumour suppressor to block the IL-6/Jak2/STAT3 signalling pathway [61]. MiR-26a, which functions as an oncogene, binds to the 3'-UTR of AP-2α and then inhibits AP-2α expression to promote glioma cell chemoresistance, migration, invasion, and proliferation [62]. MiR-6743-5p regulates the activity of STAT3 and promotes cell proliferation by directly targeting a gene associated with retinoid interferon-induced mortality-19 (GRIM-19), which serves as a tumour suppressor [63].

Exosomes, containing mRNAs, microRNAs, and long noncoding RNAs, deliver their contents to recipient cells and function as regulators of intercellular communication [64]. Based on RT-qPCR assays, hypoxic glioma-derived exosomes (H-GDEs) and the CSF of GBM patients had a significantly higher expression level of miR-1246 than normoxic glioma-derived exosomes (N-GDEs) and the CSF of low-grade glioma (LGG) patients. H-GDE-derived miR-1246 contributed to M2 macrophage polarization, which then promoted glioma progression in vivo and promoted glioma cell migration, invasion, and proliferation in vitro. Regarding the mechanism by which H-GDE-derived miR-1246 induced M2 macrophage polarization, it was found that it targeted telomeric repeat binding factor 2 interacting protein (TERF2IP) and suppressed TERF2IP expression to activate the STAT3 signalling pathway and inhibit NF-κB signalling in macrophages. In summary, H-GDE miR-1246 plays an important role in M2 macrophage polarization [65]. GBM can be subclassified into three clinically relevant types: proneural (PN), neural, mesenchymal (MES) and classical GBM [66]. MiRNAs are also involved in the process of maintaining the phenotype of GBM. A study found that the activated wnt/b-catenin signalling pathway can increase the expression of miR-125b and miR-20b, which then inhibit the expression of frizzled receptor 6 (FZD6) and ALDH1A3 to sustain Wnt/b-catenin signalling in PN GBM. However, in MES GBM, FZD6 downregulated the canonical wnt/b-catenin signalling pathway by activating the CaMKII–TAK1–NLK pathway, which can promote the STAT3 and NF-kB signalling pathways [67].

### MiRNAs regulated by STAT3

As a member of the STAT protein family, STAT3 acts as an important transcription factor, which can initiate the transcription of downstream target genes. For example, chromatin immunoprecipitation (ChIP) analysis has shown that STAT3 binds to the miR-21 promoter region to regulate the expression of miR-21; thus, IFN-β can downregulate miR-21 transcription [68]. There is a feedback loop in which miR-21 can suppress both PIAS3 protein and mRNA expression to activate STAT3 in medulloblastoma [69]. RT-PCR experiments have indicated that the expression of miR-21 increased with glioma pathological grade. Studies have found that miR-21 can target reversion-inducing cysteine-rich protein with kazal motifs (RECK) to regulate glioma cell invasion and the expression of MMP2/9 [70]. Additionally, miR-21 promoted glioma cell growth and the expression of human telomerase reverse transcriptase (hTERT) in a STAT3-dependent manner [71]. Moreover, the expression of miR-182-5p was markedly increased and positively correlated with the expression of activated STAT3 in glioma cell lines. Functional experiments have shown that miR-182-5p promotes glioma cell growth, migration, and invasion. In terms of the mechanism, STAT3 can directly interact with the miR-182-5p promoter region, and miR-182-5p can directly target sequences in the 3'-UTR of protocadherin 8 (PCDH8) [72]. In addition, in hypoxic extracellular vesicles (EVs) of glioma stem-like cells (GSCs), RT-PCR assays have shown that miR-30b-3p is expressed at high levels. Studies have found that miR-30b-3p can bind to sites on the 3′-UTR of ras homologue family member B (RHOB), decrease cell cycle arrest and inhibit cell apoptosis induced by TMZ. As a translation factor, STAT3 can bind to the miR-30b promoter, but STAT3 must form a complex with HIF1α to induce miR-30b-3p expression in GSCs under hypoxic conditions [73]. In contrast, STAT3 can inhibit the expression of some miRNAs. Upon activation of RTK signalling, STAT3 directly interacts with the miR-218 locus and then inhibits its expression. However, miR-218, acting as a tumour suppressor, can directly interact with platelet-derived growth factor receptor α (PDGFRα) to inhibit the activity of RTK signalling. In summary, activated RTK signalling increases the expression of STAT3 and then represses miR-218 expression [74]. Additionally, STAT3 can bind to the first intron of the miR-34a genomic region to suppress miR-34a expression, while miR-34a acts as a tumour suppressor in hypoxia-induced glioma cells [75].

### Other miRNAs

In addition to the above three miRNAs, other miRNAs can also affect the expression of STAT3, but the specific mechanism has not yet been elucidated. A study has shown that overexpression of miR-184 promotes cell proliferation and invasion through the JAK2/STAT3 signalling pathway [76]. Moreover, the overexpression of miR-203 increased malignant glioma (MG) cell radiosensitivity by downregulating the expression of the JAK/STAT3 pathway [77]. RT-qPCR assays revealed that miR-31 expression was significantly decreased in U87 and U251 cells. Overexpression of miR-31 induces GBM cell apoptosis and suppresses the activity of STAT3 [78]. MiR-27b, possibly mediated by the β-catenin/Tcf-4 pathway, can promote glioma cell proliferation and invasion and decrease the cell apoptosis rate [79]. In extensively mutated U87MG, miR-302/367 overexpression can clearly suppress the expression of transformation-related proteins, including STAT3 signalling [80].

In summary, some miRNAs serve as target genes and are transcriptionally regulated by STAT3. Meanwhile, to regulate the expression and activation of STAT3 in glioma, the main mechanism underlying miRNA function is through direct binding of the 3′-UTR of the target mRNA. In addition to the traditional mechanism, miRNAs can be present in exosomes to induce M2 macrophage polarization and maintain the GBM phenotype. However, some miRNAs need more in-depth study to uncover their specific mechanism. In addition, studies should focus on investigating other mechanisms of miRNA function. MiRNAs can bind to the 5′-UTR of the target gene and promote its transcription. For example, a study showed that miR-27a-3p could act as an oncogene by directly interacting with the 5'-UTR of cyclin D1 to enhance its expression in bladder cancer [81].

## LncRNAs are involved in the STAT3 signalling pathway

**Fig. 2:**
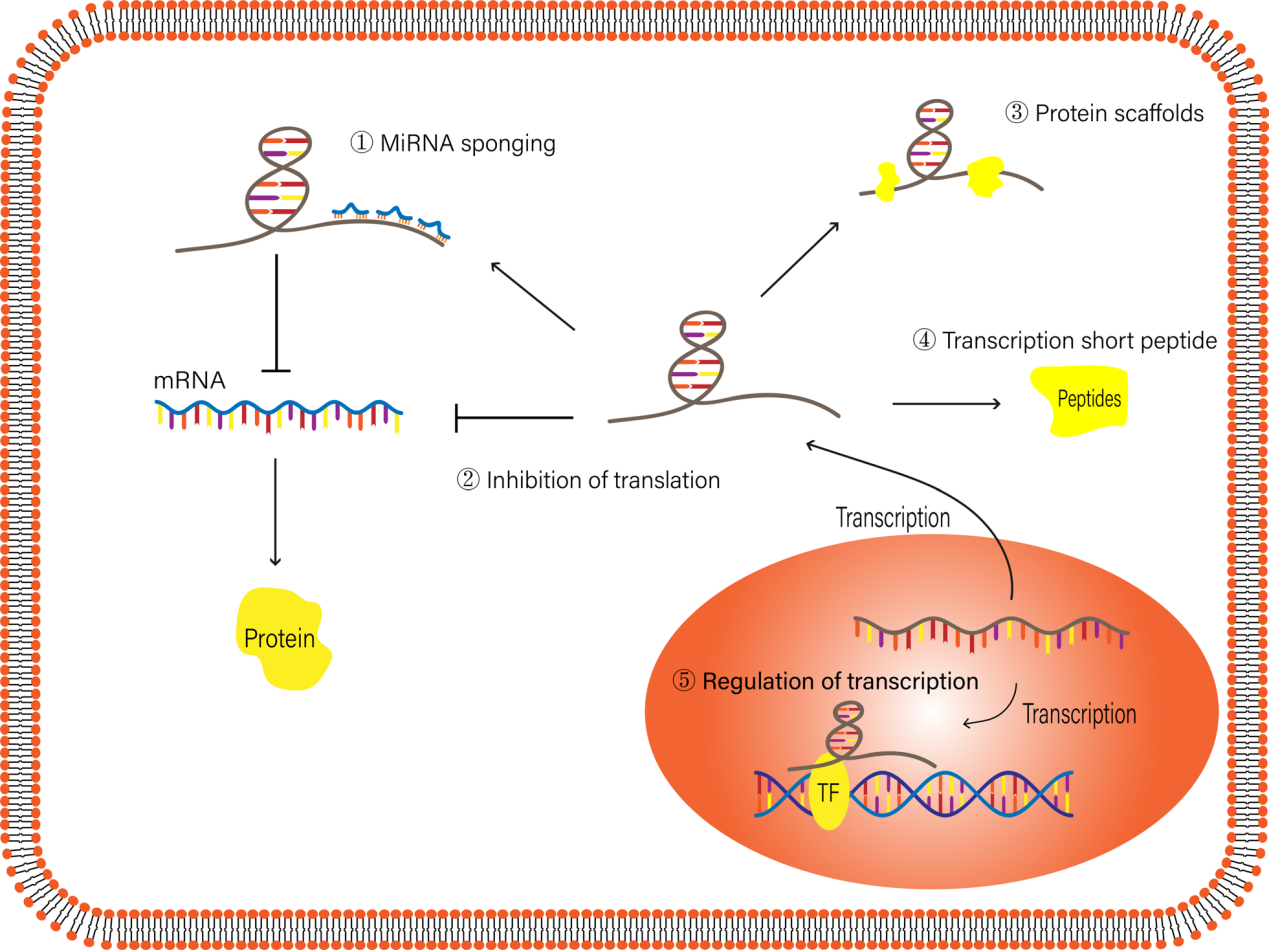
Regulatory mechanisms of lncRNAs. The regulatory molecular mechanisms of lncRNAs. In the cytoplasm, lncRNAs sponge miRNAs, regulate transcription, transcribe short peptides or serve as protein scaffolds. In the nucleus, lncRNAs regulate transcription

Long noncoding RNAs, which are more than 200 nucleotides in length, generally have no protein coding ability or can only code a few short peptides. LncRNAs are widespread in human organisms and are essential for human gene expression regulation and physiological and pathological processes. Most types of lncRNAs are transcribed by RNA polymerase II and are then capped at the 5' end, polyadenylated at the 3' end and edited through a series of splicing processes that occur in the nucleus. In addition, there are other mechanisms involved in the lncRNA maturation process. According to the position and directional relationship with the gene encoding protein, lncRNAs can be divided into five categories: sense lncRNAs, antisense lncRNAs, intronic lncRNAs, bidirectional lncRNAs, and intergenic lncRNAs [82]. In terms of the multiple functions of lncRNAs, in the cytoplasm, the most common function of lncRNAs is their ability to act as competing endogenous RNAs (ceRNAs), and lncRNAs can regulate translation, serve as protein scaffolds and transcribe short peptides [83]. In the nucleus, lncRNAs can interact with transcription factors and suppress gene transcription [84]. Similar to miRNAs, lncRNAs are also cell-specific and sequentially expressed. However, unlike miRNAs, lncRNA sequences are less conserved, and lncRNAs can fully regulate gene expression at the epigenetic level, transcription level and posttranscriptional level (Fig. 2).

Numerous studies have proven that there are a large number of abnormally expressed lncRNAs in gliomas, and they play a vital role in the occurrence and development of gliomas. The abnormal expression and role of these lncRNAs in gliomas makes them promising targets for the early diagnosis and treatment of gliomas. LncRNAs regulate the expression and activation of various signalling pathways and target genes in gliomas [85]. Among them, lncRNAs related to STAT3 are mainly divided into the following types as shown in Table 2.

**Table 2.** LncRNAs/CircRNAs involved in STAT3 signaling pathway

LncRNA/CircRNA	Up-/down-regulation	Role in glioma	Biological functions	Target gene	Refs.
CASC9	Up	Oncogene	Promote invasion and proliferation	miR‐519d/STAT3	[86]
CRNDE	Up	Oncogene	Promote proliferation, migration, and invasion	miR-384/PIWIL4	[87]
LINC00115	Up	Oncogene	Promote proliferation	miR-200b, miR-200c/ZNF596	[88]
miR155HG	Up	Oncogene	Promote proliferation	miR-185-5p/ANXA2	[89]
PEG10	Up	Oncogene	Promote proliferation, migration, and invasion	miR-506	[90]
GHET1	Up	Oncogene	Promote proliferation, migration, and invasion	miR-216a	[91]
circ-HIPK3	Up	Oncogene	Promote proliferation, migration, and invasion	miR-124-3p/STAT3	[102]

### LncRNAs regulate the expression and activation of STAT3 through the ceRNA mechanism

Their ability to act as competing endogenous RNAs (ceRNAs) or miRNA sponges is the most common function of lncRNAs. The lncRNAs located in the cytoplasm likely regulate the expression of downstream genes through this ceRNA mechanism. The expression of lncRNA CASC9 (cancer susceptibility candidate 9) is significantly increased in glioma tissue and is positively related to advanced pathological grade of glioma. Knocking down lncRNA CASC9 inhibits glioma cell invasion and proliferation. In terms of the mechanism responsible for this effect, the 3′-UTR of CASC9 can interact with miR‐519d, which acts as a tumour suppressor by directly targeting the 3′‐UTR of STAT3 mRNA, to increase the expression of STAT3. Moreover, ChIP and luciferase reporter assays demonstrated that STAT3 can increase the expression of CASC9 by binding to the promoter of CASC9. Taken together, in glioma, lncRNA CASC9 increases STAT3 expression by sponging miR‐519d, while STAT3 interacts with the CASC9 promoter to accelerate CASC9 expression [86].

### LncRNAs regulate the expression of genes upstream of STAT3

The activation of STAT3 is precisely regulated. In normal cells, the activation of STAT3 is short-lived, while in glioma cells, STAT3 exhibits constitutively high activation and participates in the occurrence and development of tumours due to the lack of upstream regulatory signals. Similar to miRNAs, lncRNAs can also increase the expression and activity level of STAT3 by regulating its upstream regulatory signals. Coimmunoprecipitation and immunoprecipitation experiments have shown that piwi-like RNA-mediated gene silencing 4 (PIWIL4), belonging to the PIWI subfamily, could induce activation of STAT3. The expression of colorectal neoplasia differentially expressed (CRNDE) is high in glioma. Knockdown of CRNDE induced glioma cell apoptosis, while overexpression of CRNDE promoted U87 and U251 cell proliferation, migration and invasion. RT-PCR assays showed that the expression of miR-384 was decreased in glioma tissues and glioma cell lines and negatively correlated with advanced glioma pathological grade. Luciferase assays showed that CRNDE could bind to miR-384, while miR-384 could target PIWIL4 to function as a tumour suppressor. LncRNA CRNDE increased PIWIL4 expression to induce p-STAT3 through a ceRNA mechanism [87]. In addition, Kaplan–Meier survival analysis and datasets showed that the expression level of LINC00115, which is activated by TGF-β, was higher in GBM than in LGG and correlated with a statistically poor prognosis. In vitro experiments found that knockdown of LINC00115 inhibited GSC proliferation and neuro-like sphere formation. In vivo experiments found that LINC00115 knockdown reduced glioma tumour growth and prolonged the survival of animals. LINC00115 can physically associate with miR-200b and miR-200c to competitively inhibit the function of miR-200b and miR-200c. MiR-200b and miR-200c can bind to the 5′-UTR of zinc finger protein 596 (ZNF596) mRNA. Meanwhile, ChIP-qPCR and luciferase reporter analysis demonstrated that ZNF596 could directly bind with the promoter of enhancer of zeste homologue 2 (EZH2), which is a lysine methyltransferase and the enzymatic component of the polycomb repressive complex 2. Due to EZH2 promoting histone H3 lysine 27 trimethylation (H3K27me3) and activating STAT3 signalling, LINC00115, acting as an oncogene, competitively binds with miR-200 s and activates ZEB1 signalling, and ZNF596 enhances EZH2/STAT3 signalling [88]. Moreover, based on three public human astrocytoma databases (TCGA, CGGA and Rembrandt), the expression of the miR155 host gene (miR155HG) is high in GBM tissues and positively associated with the expression of genes that reduce apoptosis and cell death. The GBM mouse model found that miR155HG increased tumour volume. In vitro, miR155HG silencing inhibited cell proliferation and increased cell apoptosis. These results show that miR155HG is an oncogene in GBM. In terms of the mechanism, miR155HG can bind with miR-185-5p and then sponge miR-185-5p to downregulate its expression. MiR-185-5p directly targets the 3′-UTR of ANXA2, which can act as an oncogene by regulating the expression of p-STAT3. Additionally, ChIP and luciferase reporter gene assays showed that p-STAT3 could bind to the promoter region of miR155HG to increase the expression of miR155HG. In summary, the authors established the miR155HG/miR-185-5p/ANXA2 loop in GBM [89].

### LncRNAs regulated by STAT3

Similar to miRNAs, lncRNAs can also serve as target genes of STAT3 in gliomas and undergo transcriptional regulation. Previous studies have shown that STAT3 interacts with the promoter region of CASC9, while CASC9 competitively binds with miR‐519d to increase the expression of STAT3. Similarly, p-STAT3 directly binds to the miR155HG promoter region. MiR155HG inhibits miR-185-5p expression through a ceRNA mechanism and then promotes ANXA2 expression to accelerate the activation of STAT3.

### LncRNAs regulate STAT3 through an unknown mechanism

In addition, some lncRNAs can affect the expression of STAT3 without a clear and specific mechanism. For example, knockdown of lncRNA PEG10 inhibited U251 cell proliferation, migration, and invasion and inactivated the Raf/MEK/ERK and JAK1/STAT3 signalling pathways by increasing miR-506 expression [90]. In addition, lncRNA gastric carcinoma highly expressed transcript 1 (GHET1), acting as an oncogene; promoted cell proliferation, migration, and invasion; and activated the JAK2/STAT3 and p53/survivin signalling pathways by miR-216a downregulation [91].

In summary, although lncRNA PEG10 and GHET1 require future studies to reveal the specific mechanism, it is clear that the ceRNA mechanism plays an important role in the regulation of the STAT3 pathway by lncRNAs. Moreover, studies established 2 positive feedback loops: the miR155HG/miR-185-5p/ANXA2 loop and the CASC9/miR‐519d/STAT3 loop. Regarding transcription factor research in glioma formation and progression, future studies can also try to verify the existence of such a positive feedback loop. For instance, Lin and colleagues, found that the positive feedback loop HOXC-AS2/miR-876-5p/ZEB1 could regulate epithelial–mesenchymal transition (EMT) in GBM [92]. In addition to the ceRNA mechanism, research should focus on additional mechanisms. In glioma, lncRNA PLAC2 negatively regulates RPL36 expression levels through the STAT1 signalling mechanism. In the nucleus, PLAC2 can bind with STAT1 and then interact with the promoters of RPL36, while cytoplasmic PLAC2 can also inhibit STAT1 translocation into the nucleus [93].

## CircRNAs are involved in the STAT3 signalling pathway

**Fig. 3:**
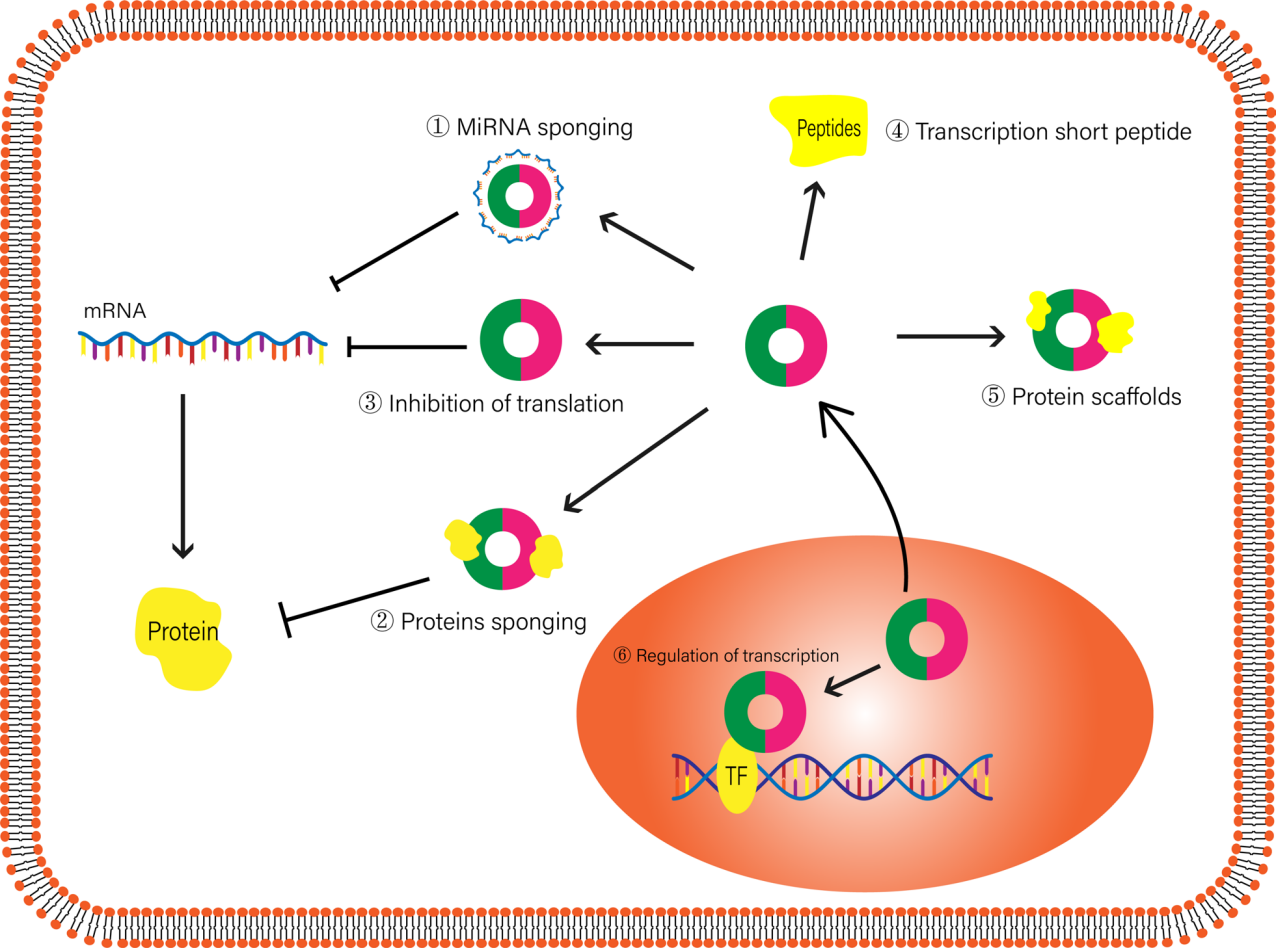
Regulatory mechanisms of circRNAs. The regulatory molecular mechanisms of circRNAs. In the cytoplasm, circRNAs sponge miRNAs and proteins. CircRNAs regularly transcribe short peptides or serve as protein scaffolds. In the nucleus, circRNAs regulate transcription

Following the investigation of miRNAs and lncRNAs, circRNAs have become a new research focus. Unlike linear RNA, circRNAs have a covalent single-stranded closed loop structure, lacking a 5' cap and 3' tail, and the structure of circRNA is highly stable compared with linear RNA. According to the various positions and formation mechanisms of circRNAs, they can be divided into three subclasses: exonic circRNAs, intronic circRNAs and EIciRNAs [94]. It has been found that more than 80% of circRNAs are derived from protein-coding gene exons in the cytoplasm. In the past, it was thought that circRNAs were a by-product of transcription, had low expression abundance and were non-functional. However, with the development of high-throughput sequencing and RNA detection technology, a large number of circRNAs, which are produced by back-splicing of a pre-mRNA, have been found in normal tissues and different tumours. The function and molecular mechanism of circRNAs have also been revealed. Studies have found that circRNAs work through various mechanisms. In the cytoplasm, circRNAs can regulate miRNAs through ceRNA mechanisms. CircRNAs can also form circRNA-protein complexes (circRNPs) by binding to proteins, thereby regulating the function of related proteins, intracellular localization and transcription of related genes. Some circRNAs can transcribe short peptides or serve as protein scaffolds [95, 96]. In the nucleus, circRNAs can regulate the transcription and splicing of genes [97] (Fig. 3).

The expression of circRNAs is tissue specific and developmental stage dependent. Many studies have shown that abnormally expressed circRNAs are involved in the pathophysiological process of glioma. For example, the circRNA HIPK3 [98] regulates the proliferation, migration, and invasion of tumour cells through a ceRNA mechanism. Circ-DICER1 [99] is involved in the angiogenesis of glioma. Circ_0076248 [100] is involved in the immune response of glioma. Circ_0001649 can be used as a diagnostic and prognostic indicator [101].

There is only one study on the role of circRNA in regulating the STAT3 pathway. Based on qRT-PCR assays, the expression of the circular RNA HIPK3 was significantly higher in the U251 and U87 cell lines than in the normal cell line (HEB), while miR-124-3p was expressed at a low level in the U251 and U87 cell lines. Functional experiments confirmed that circ-HIPK3 knockdown by targeted siRNAs inhibited cell proliferation, migration, and invasion and induced cell apoptosis. Mechanistic experiments have shown that circ-HIPK3 directly binds to miR-124-3p and that miR-124-3p can directly target the 3'-UTR of STAT3 mRNA. In summary, circ-HIPK3 suppresses the mRNA expression of miR-124-3p, an inhibitor of STAT3, through a ceRNA mechanism. Due to the low expression of miR-124-3p, STAT3 expression is increased [102].

In summary, studies should investigate more circRNAs involved in the glioma STAT3 signalling pathway. Mechanistically, similar to lncRNAs, the ceRNA mechanism is the most common functional mechanism of circRNas, while few studies have found that circRNAs can induce STAT3 nuclear translocation. In colorectal cancer, circSPARC not only regulates the expression of JAK2 through the ceRNA mechanism to affect the activation of STAT3 but also enhances the nuclear translocation of p-STAT3 through the recruitment of FUS RNA binding protein [103].

## Conclusions

**Fig. 4:**
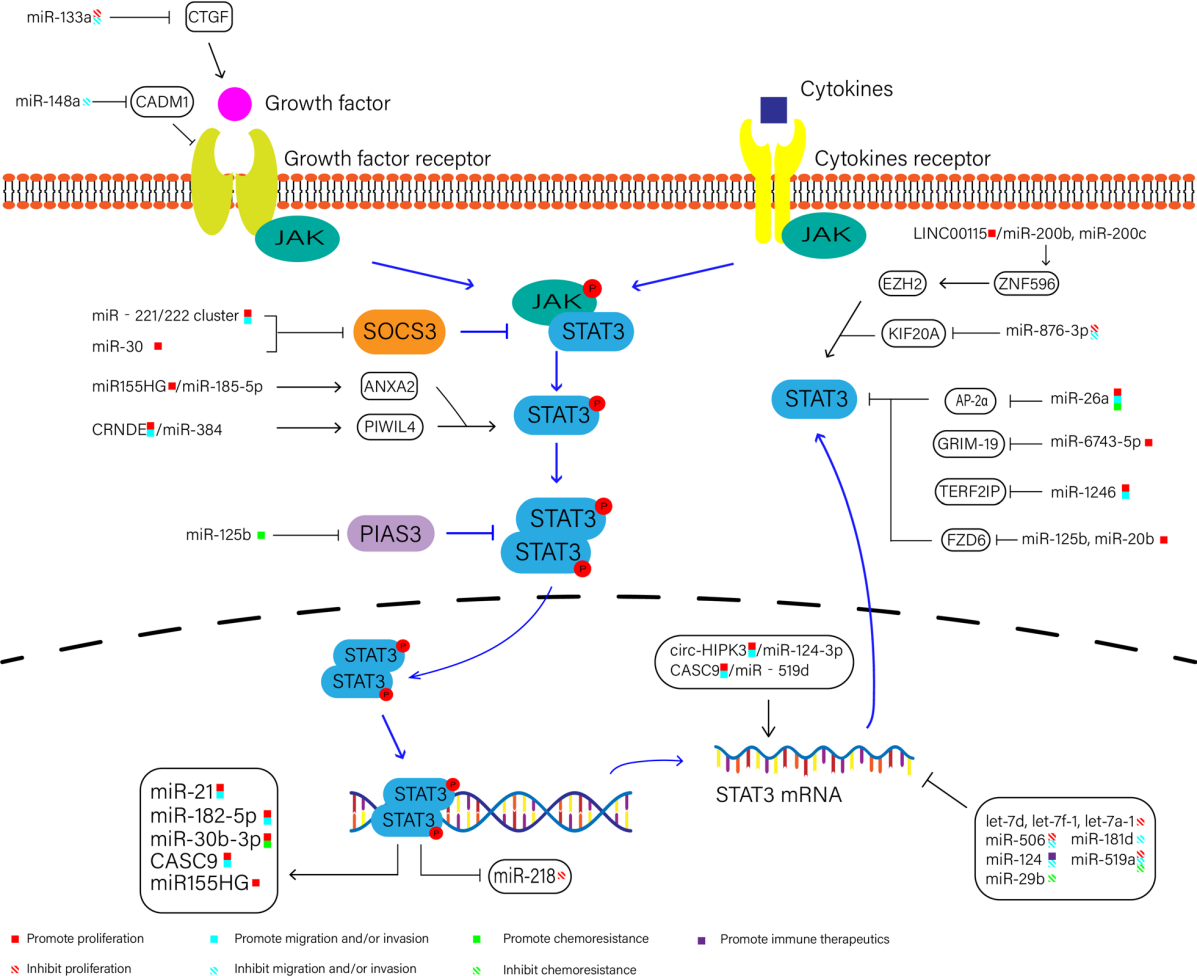
NcRNAs involved in the STAT3 pathway in glioma. NcRNAs and their targets involved in the STAT3 pathway regulate the formation and development of glioma. In glioma, the activation of STAT3 can be induced by growth factors and cytokines. Cytokine or growth factor binding to its receptor can activate JAK through tyrosine phosphorylation. Under the action of JAKs, STAT3 can be activated and form homodimers or heterodimers (STAT1/3) and then enter the nucleus to function as a transcription factor. NcRNAs can regulate the expression and activation of STAT3 or be regulated by STAT3. The figure shows the specific ncRNAs with clear mechanisms mentioned in the text

In summary, an increasing number of studies have shown that noncoding RNAs play an important role in a variety of malignant tumours. The role and mechanism of noncoding RNAs in glioma has been a research hotspot in recent years, and they are expected to become diagnostic and prognostic markers and therapeutic targets for glioma. Moreover, the continuous high expression and activation of STAT3 is an important factor in the occurrence and development of glioma. STAT3 is considered a potential therapeutic target for glioma, but there is no effective targeted STAT3 clinical treatment method for glioma. In this review, we analysed the noncoding RNAs that regulates the expression of STAT3 and that are regulated by STAT3 in gliomas (Fig. 4).

In terms of STAT3, as a transcription factor, there is no doubt that STAT3 can play an important role in regulating the expression of ncRNAs. LncRNAs CASC9 and miR155HG promote STAT3 through the ceRNA mechanism, and STAT3, in turn, acts as a transcription factor in the nucleus of glioma cells to promote the expression of CASC9 and miR155HG. These molecules form a positive feedback loop to promote their expression. For miRNAs, activated STAT3 can promote the expression of many oncogenic miRNAs, such as miR-182-5p, miR-21 and miR-30b-3p. Similarly, STAT3 can also inhibit the expression of miR-218, a tumour suppressor. In terms of miRNAs, the most common mechanism of miRNA function is binding to the 3′-UTR of the target mRNA. In the STAT3 signalling pathway of glioma, miRNAs can directly target STAT3 mRNA, genes closely related to STAT3 activation (PIAS3, SOCS3), and upstream genes of STAT3. In addition, miRNAs, such as miR-1246, can also play a role by being packaged inside exosomes. However, some miRNAs can regulate the expression and activation of STAT3, but the specific mechanism is still not clear; thus, it is necessary to explore these specific mechanisms. In terms of lncRNAs and circRNAs, these RNAs can regulate the expression of target genes at the epigenetic, transcriptional and posttranscriptional level. However, studies have mainly focused on lncRNAs and circRNAs that regulate the transcription level of STAT3 through the ceRNA mechanism. In addition to the ceRNA mechanism, other mechanisms of lncRNAs and circRNAs have been reported. For example, in nasopharyngeal carcinoma, lncRNA differentiation antagonizing nonprotein coding RNA (DANCR) can interact with STAT3 to enhance JAK1 binding to STAT3 [104]. LncRNA LOC101927514 was detected in the nucleus where it interacted with STAT3, thereby participating in the release of IL-6 and IL-8 [105]. Circ-Amotl1 not only interacts with STAT3 to increase STAT3 expression but also promotes STAT3 nuclear translocation [106]. Therefore, in addition to ceRNA mechanisms, studies should explore other mechanisms by which lncRNAs and circRNAs regulate the STAT3 signalling pathway in gliomas.

In recent years, research on tumour immunity has gained more attention. Interferon, as an immune-related cytokine, can play an important role in the antitumour process, and interferons play important roles in STAT3 activation. Of course, some ncRNAs are also involved in this process. In glioma, IFN-β regulates miR-21 through STAT3, which is an interesting finding. IFN-β can induce STAT3 activation, but the role of STAT3 is distinct. The p-STAT3 induced by IFN-β can directly bind to the promoter of miR-21 and then inhibit the transcription of this carcinogenic miRNA, but STAT3 promotes the transcription of miR-21 without IFN-β [68]. Different cytokine stimuli and cell types may contribute to the controversial role of STAT3. Although there is no research on the lncRNAs and circRNAs involved in this process of glioma, their mechanisms are different from those of miRNAs. In the cytoplasm of hepatocellular carcinoma cells, LncRNA00364, which is upregulated by IFN-γ can directly interact with STAT3 and then inhibit the phosphorylation of tyrosine-705 of STAT3 to function as a tumour suppressor [107]. In addition, immune cells involved in the tumour microenvironment also play important roles in tumour immunity. Some ncRNAs are involved in the STAT3 signalling pathway of immune cells. M2 polarization macrophages make up a majority of glioma tumour-associated macrophages and help create a tumour-immunosuppressive microenvironment to promote tumour function [108]. To induce M2 macrophage polarization, H-GDE-derived miR-1246 can inhibit TERF2IP expression and then activate the STAT3 signalling pathway in macrophages [65]. Contrary to M2 macrophages, T cells can promote tumour suppression. In glioma, miR-124 can enhance T cell-mediated immune clearance and modulate T helper cell differentiation by inhibiting the STAT3 signalling pathway [48]. As the literature has not revealed relevant findings, glioma-related studies should focus more attention on lncRNAs and circRNAs involved in the STAT3 signalling pathway of immune cells. MHC class I-related chain (MIC)-A, which is a downstream target of STAT3, can activate natural killer T (NKT) cells. In cervical cancer, a study has shown that LINC00240 can promote STAT3 expression by sponging miR-124-3p, and then STAT3 can inhibit MICA to reduce the cytotoxicity of NKT cells [109]. Overall STAT3 plays a vital role in tumour immunity. To reveal the role of STAT3 in glioma, future studies should focus on the ncRNAs involved in these two processes.

Therefore, further exploration of noncoding RNAs related to STAT3 expression and activation in glioma may provide an avenue for STAT3-targeted glioma treatment.

## Data Availability

Not applicable.
